# Saliva and Serum Immune Responses in Apical Periodontitis

**DOI:** 10.3390/jcm8060889

**Published:** 2019-06-21

**Authors:** Milla Pietiäinen, John M. Liljestrand, Ramin Akhi, Kåre Buhlin, Anders Johansson, Susanna Paju, Aino Salminen, Päivi Mäntylä, Juha Sinisalo, Leo Tjäderhane, Sohvi Hörkkö, Pirkko J. Pussinen

**Affiliations:** 1 Oral and Maxillofacial Diseases, University of Helsinki and Helsinki University Hospital, FI-00014 Helsinki, Finland; kare.buhlin@ki.se (K.B.); susanna.paju@helsinki.fi (S.P.); aino.m.salminen@helsinki.fi (A.S.); leo.tjaderhane@helsinki.fi (L.T.); pirkko.pussinen@helsinki.fi (P.J.P.); 2 Medical Microbiology and Immunology, Research Unit of Biomedicine, University of Oulu, FI-90014 Oulu, Finland; sohvi.horkko@nordlab.fi; 3 Medical Research Center, Oulu University Hospital and University of Oulu, FI-90014 Oulu, Finland; 4 Nordlab, Oulu University Hospital, FI-90220 Oulu, Finland; 5 Division of Periodontology, Department of Dental Medicine, Karolinska Institutet, S-141 04 Huddinge, Sweden; 6 Department of Odontology, Molecular Periodontology Research, Umeå University, S-901 87 Umeå, Sweden; anders.p.johansson@umu.se; 7 Institute of Dentistry, University of Eastern Finland, FI-70211 Kuopio, Finland; paivi.mantyla@uef.fi; 8 Kuopio University Hospital, Oral and Maxillofacial Diseases, FI-70029 Kuopio, Finland; 9 HUCH Heart and Lung Center, Helsinki University Hospital, FI-00029 Helsinki, Finland; juha.sinisalo@hus.fi

**Keywords:** apical periodontitis, adaptive immunity, saliva, serum, antibody

## Abstract

Apical periodontitis is an inflammatory reaction at the apex of an infected tooth. Its microbiota resembles that of marginal periodontitis and may induce local and systemic antibodies binding to bacteria- and host-derived epitopes. Our aim was to investigate the features of the adaptive immune response in apical periodontitis. The present Parogene cohort (*n* = 453) comprises patients with cardiac symptoms. Clinical and radiographic oral examination was performed to diagnose apical and marginal periodontitis. A three-category endodontic lesion score was designed. Antibodies binding to the bacteria- and host-derived epitopes were determined from saliva and serum, and bacterial compositions were examined from saliva and subgingival samples. The significant ORs (95% CI) for the highest endodontic scores were observed for saliva IgA and IgG to bacterial antigens (2.90 (1.01–8.33) and 4.91 (2.48–9.71)/log10 unit), saliva cross-reacting IgG (2.10 (1.48–2.97)), serum IgG to bacterial antigens (4.66 (1.22–10.1)), and Gram-negative subgingival species (1.98 (1.16–3.37)). In a subgroup without marginal periodontitis, only saliva IgG against bacterial antigens associated with untreated apical periodontitis (4.77 (1.05–21.7)). Apical periodontitis associates with versatile adaptive immune responses against both bacterial- and host-derived epitopes independently of marginal periodontitis. Saliva immunoglobulins could be useful biomarkers of oral infections including apical periodontitis—a putative risk factor for systemic diseases.

## 1. Introduction

Apical periodontitis (AP) is an inflammatory disease that affects the tissues surrounding the apex of the tooth. It is triggered by oral pathogens infecting root canal. Both acute (abscess) and chronic inflammatory reaction (periapical granuloma and radicular cyst) can develop depending on the intensity of the bacterial infection and the host immune responses. Primary apical periodontitis usually develops when the bacteria in a caries lesion enter through enamel and dentin and cause microbial colonization of the pulp and eventually necrosis of the pulp tissue. Secondary apical periodontitis arises from a persistent infection of previously treated root canals or leakage of the filling in a root canal-treated tooth. Apical periodontitis is diagnosed from radiographs as an evident radiolucent area (referred to as endodontic lesion) at the tip of the root. Even slight radiographically evident widening of the periapical space is associated with an infection in the tooth [1]. AP is treated with root canal treatment where infection is eliminated chemomechanically and the root canal is filled.

Apical periodontitis is a highly common and underdiagnosed disease. It is estimated that approximately 10% of all teeth are endodontically treated, 5% have periapical radiolucencies [2], and the prevalence of apical periodontitis varies between 24 and 86% in different populations [3]. Up to 78% of endodontically treated teeth have root canal fillings with poor quality and ~36% of the root canal-treated teeth present apical periodontitis [2], suggesting that recurrent or persistent endodontic infections are common. Apical periodontitis is usually symptomless, and it can be diagnosed only by radiography.

Endodontic infections are polymicrobial and the structure of the intracanal biofilm may evolve toward obligate aerobes and Gram-negative anaerobes as the infection progresses. More than 400 different microbial taxa have been identified in endodontic samples from teeth with different forms of apical periodontitis [4]. Several studies have also shown that distinct bacterial communities are found in primary and secondary AP [5,6,7,8]. Despite the high interindividual variability in endodontic microbial community composition, the most often encountered phyla in the intracanal samples include Firmicutes, Actinobacteria, Bacteroidetes, Proteobacteria, and Fusobacteria. Genera such as Prevotella, Fusobacterium, Parvimonas, Lactobacillus, Streptococcus, and Porphyromonas are highly prevalent in intracanal samples [9]. Several members of these genera are also considered etiological pathogens for marginal periodontitis and the microbial profiles of these two conditions resemble each other [10].

Microbial antigens stimulate innate immune responses in periapical tissue aiming to restrict the infection. The expression of proinflammatory cytokines, prostaglandins, and proteolytic enzymes are markedly increased in the areas of tissue destruction [11]. As one antimicrobial strategy, apical periodontitis is also associated with oxidative stress [12]. Some studies suggest a modest contribution of endodontic infections to the plasmatic inflammatory markers [13,14], while a recent study found a significant association between endodontic lesions and systemic inflammatory burden in young adults [15].

Additionally, adaptive immune responses are activated to prevent the microbial invasion into the tissues surrounding teeth or into circulation. High concentrations of local immunoglobulins IgG and IgA and lesser amounts of IgM and secretory IgA are present in the inflamed tissues [16,17,18,19]. The levels of systemic immunoglobulins, including total IgA, IgG, and IgM, are increased in patients with AP [13]. We recently showed that subgingival *Porphyromonas endodontalis* levels and serum IgG against it were associated with a higher endodontic lesion score [20]. Several oral pathogens are also known to be able to induce cross-reactive antibodies, which may influence inflammatory responses. The cross-reactive antibodies are part of an immunological process called molecular mimicry, in which bacterial antigens sufficiently resembling human proteins are able to induce the production of antibodies reacting with human epitopes. The most studied epitopes include those present in the heat shock proteins (HSPs) and in oxidized low-density lipoproteins (oxLDL) [21].

The association of marginal periodontitis with several systemic conditions such as cardiovascular diseases (CVDs) is well established [22]. Due to similarities in the inflammatory and microbial profiles between marginal periodontitis and AP, it is also suggested that there could be a link between AP and CVDs [23,24]. Even though the possible association of apical periodontitis with systemic diseases has been of high interest, the adaptive immune response against the disease has not been investigated in detail. In this study we aimed to investigate serum and saliva antibodies against several oral pathogens associated with apical periodontitis and the role of cross-reactive antibodies in the disease.

## 2. Experimental Section

### 2.1. Population

The Corogene is a prospective cohort of Finnish patients who had an indication to coronary angiography between June 2006 and March 2008 at the Helsinki University Hospital [25]. The present study comprises the Parogene, which is a substudy of 508 patients with clinical and radiographic oral health examinations. The details of the examinations have been described elsewhere [26]. The information of smoking habits was collected with a questionnaire before the oral examination. The presence of diabetes (type I and II) was obtained from medical records. All subjects signed an informed consent and the study was approved by the Helsinki University Hospital ethics committee (approval reference number 106/2007). Patients with antibody measurements from serum and saliva samples were included (*n* = 453, 89.2% of the whole cohort). The number of dentate patients and subgingival samples was 426 (n of edentulous 27, 6.0%).

### 2.2. Oral Diagnosis

Endodontic lesions were diagnosed from the radiographs as described in detail earlier [20]. The recorded findings included root canal fillings, widened periapical space indicating irreversible pulpitis or precursors for endodontic lesions [1], and apical periodontitis seen as periradicular destruction in the tip of the root. An endodontic lesion score was defined to describe the severity of apical periodontitis [20]. Score I included patients without endodontic lesions (*n* = 162, 38.2%); score II, patients with ≥1 widened periapical space and/or 1 tooth with apical periodontitis (*n* = 194, 45.2%); and score III, patients with ≥2 teeth with apical periodontitis (*n* = 68, 16.0%). In addition, another subgrouping—the endodontic treatment score—was designed according to treated/untreated apical periodontitis: I, no endodontic lesions (*n* = 352, 77.7%); II, teeth with apical periodontitis, all with root canal fillings (*n* = 51, 11.3%); and III, apical periodontitis in tooth/teeth without root canal fillings (*n* = 50, 11.0%). Number of teeth and implants, presence of carious teeth, and inadequate root canal fillings were also recorded from the radiographs.

Diagnosis for marginal periodontitis was based on alveolar bone loss (ABL) detected in the radiographs and bleeding on probing (BOP) registered in the clinical examination from four sites of each tooth. Patient was considered periodontally healthy, when no ABL and <25% BOP was present; with gingivitis, when no ABL but ≥25% BOP; and with periodontitis, when ABL was present [27].

### 2.3. Bacterial Analyses

Subgingival plaque samples were collected from the deepest pathological periodontal pocket (≥ 4 mm) in each dentate quadrant as described earlier [28]. The microbiome analysis including 79 taxa was performed by using the checkerboard DNA-DNA hybridization assay [29] and the data was analyzed as described in our earlier article [28]. In the present work, we summed up the results of Gram-positive taxa (*n* = 45) and Gram-negative taxa (*n* = 34), which are presented in Supplementary Table S1.

Saliva samples were collected after stimulation by chewing for 5 min, and a minimum of 2 mL of saliva was collected by expectoration. The methods for sample processing and quantitative real-time PCR have been described in detail earlier [30]. Saliva concentration of four bacterial species associated with periodontitis was analyzed: *Aggregatibacter actinomycetemcomitans*, *Porphyromonas gingivalis*, *Tannerella forsythia*, and *Prevotella intermedia*.

### 2.4. Antibody Determinations

Serum IgA- and IgG-class antibody levels against seven bacterial species—*A. actinomycetemcomitans*, *P. gingivalis*, *T. forsythia*, *P. intermedia*, *Campylobacter rectus*, *Fusobacterium nucleatum*, and *P. endodontalis*—were determined with ELISA as described earlier [31]. The antigens were composed of formalin-killed whole cells and two dilutions in duplicate were measured [32]. After all antibody levels were determined, the absorbances were normalized according to the reference applied on each plate and the results were expressed as continuous ELISA-units (EU). The list of the antigens, sample dilutions, and coefficients of interassay variations are presented earlier [31].

Saliva IgA- and IgG-class antibody levels against five species—*A. actinomycetemcomitans*, *P. gingivalis*, *T. forsythia*, *P. intermedia*, *and P. endodontalis*—were determined from saliva supernatants obtained after centrifugation at 9300× *g* for 3 min. The target antigens used in the assays were either heat-killed whole bacterial cells or oxidized LDL epitope malondialdehyde acetaldehyde modification (MAA-LDL), copper-oxidized LDL (CuOx-LDL) [33], recombinant *P. gingivalis* virulence factor gingipain (Rgp44) [34], and 60-kDa *A. actinomycetemcomitans* heat shock protein (Aa-HSP60) [35]. Levels of salivary IgA and IgG antibodies to oxidized LDL and bacterial epitopes were determined by chemiluminescence immunoassay as previously described in detail [36,37]. The saliva samples were diluted accordingly: 1:250 for total IgA and IgG, 1:50 for IgA to oxidized antigens, 1:20 for Aa-HSP60, and 1:10 for bacterial antigens. For IgG measurements, saliva samples were diluted 1:10 for all antigens. Each saliva sample was measured as triplicates. Immunoassay results were presented as relative light units (RLU) per 100 milliseconds (ms).

### 2.5. Calculations of Cross-Reactive Antibodies and Antibodies Binding to Bacterial Antigens

In addition to the mean levels of antibodies against each specific antigen, the combined antibody levels of saliva and serum IgA and IgG were calculated. The bacterial antigens included *A. actinomycetemcomitans*, *P. gingivalis*, *P. intermedia*, *P. endodontalis*, and *T. forsythia* (referred as IgA/IgG against bacteria). The epitopes recognized in *P. gingivalis* and *A. actinomycetemcomitans* giving rise to cross-reactive antibodies with MAA-LDL included Rgp-44 and Aa-HSP60 (referred as cross-reacting IgA/IgG).

### 2.6. Statistical Methods

The characteristics are presented as mean values with standard deviations (SD) or 95% confidence intervals. For clarity, standard error is displayed in the figures as error bars. In the supplementary tables, the bacterial levels are presented as medians and interquartile ranges (IQR). Before statistical comparisons, the antibody and bacterial levels were transformed with 10-base logarithm. The significance of the differences was tested by using *t*-test, ANOVA, Chi-square, or Mann–Whitney, when appropriate. The weighted linear terms were examined with ANOVA and Jonckheere–Terpstra test for normally distributed and skewed data, respectively. The associations were analyzed by using linear and logistic regression models adjusted for age, sex, marginal periodontitis (healthy, gingivitis, and periodontitis), number of teeth, and smoking (never/ever). When the dependent variable was composed of several subgroups, multinomial regression was used. When the associations were examined in the subgroup of patients without marginal periodontitis, the confounders were limited to age, sex, and smoking (never/ever).

## 3. Results

Characteristics of the dentate population are presented in Table 1. The mean (SD) age was 62.9 (9.1) years and 67% were males. The mean number of teeth was 21.4 (7.5), and caries and apical periodontitis were common findings, in 47.4% and 23.8% of the population, respectively. Also, marginal periodontitis ranging from mild to severe was present in most patients (75.5%).

The endodontic findings registered included root canal fillings, widened periapical space, and apical periodontitis. Mean antibody levels in serum and saliva against specific antigens, as well as the saliva and subgingival bacterial levels according to the endodontic findings are presented in supplementary tables (Supplementary Tables S2 and S3).

Among serum or saliva IgA-class antibody levels only sporadic significant differences were observed between patients with and without endodontic findings, whereas among IgG-class antibodies several significant differences were found. The antigens producing these differences included *A. actinomycetemcomitans*, *P. gingivalis*, *P. intermedia*, *P. endodontalis*, *C. rectus*, *F. nucleatum*, and *T. forsythia*, as well as Aa-HSP60, rgp44, MAA-LDL, and CuOx-LDL (Table S2). Among the salivary or subgingival bacterial concentrations, significant differences were mostly found between patients with and without widened periapical spaces (Table S3).

**Figure 1:**
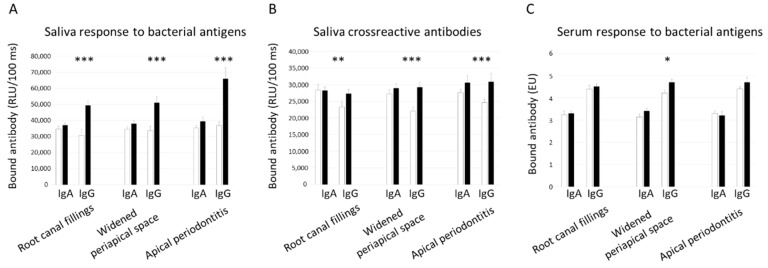
Saliva and serum antibody levels according to endodontic findings. The patients were divided into groups according to the presence of root canal fillings, widened periapical spaces, and apical periodontitis. Saliva (A, B) and serum (C) IgA- and IgG-class antibodies were determined. The bacterial antigens included *A. actinomycetemcomitans*, *P. gingivalis*, *P. intermedia*, *P. endodontalis*, and *T. forsythia*. The antigens giving rise to cross-reactive antibodies included MAA-LDL, Rgp-44, and Aa-HSP60. White columns depict the absence of the endodontic finding and black columns depict the presence of the endodontic finding. Means and standard errors are shown. The asterisks depict statistical significance between the groups defined by the *t*-test after logarithmic transformation: * *p* < 0.05, ** *p* < 0.01. *** *p* < 0.001.

**Figure 2:**
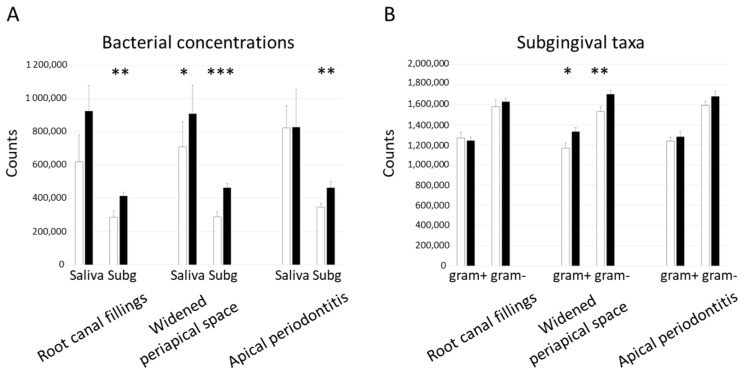
Saliva and subgingival bacteria according to endodontic findings. The patients were divided into groups according to the presence of root canal fillings, widened periapical spaces, and apical periodontitis. Salivary bacterial concentrations of *A. actinomycetemcomitans*, *P. gingivalis*, *P. intermedia*, and *T. forsythia* were determined by qPCR, and subgingival *A. actinomycetemcomitans*, *P. gingivalis*, *P. intermedia*, *P. endodontalis*, and *T. forsythia* by checkerboard DNA–DNA hybridization (A). This method was also used to examine subgingival 79 taxa, which were divided into Gram-positive (*n* = 45) and Gram-negative (*n* = 34) (B). The white columns depict the absence, and black columns presence of the endodontic finding. Means and standard errors are shown. The asterisks depict statistical significance between the groups defined by the *t*-test after logarithmic transformation: * *p* < 0.05, ** *p* < 0.01. *** *p* < 0.001.

For further analyses, the microbial biomarkers were combined, and the mean levels are presented in Figure 1 and Figure 2. The combinations included antibody level against bacteria, cross-reactive antibodies, salivary bacteria, and subgingival bacteria. Similarly as above, the mean saliva IgG-class antibody levels against bacteria and the cross-reactive antibodies as well as saliva and subgingival bacterial levels were higher in patients with endodontic findings. From the serum antibody levels, the IgG against bacteria were higher only in patients with widened periapical spaces (*p* = 0.015). In these patients, the increase of subgingival bacterial levels was due to both Gram-positive (*p* = 0.022) and Gram-negative (*p* = 0.005) species.

The associations of antibody and bacterial levels with endodontic findings are presented in Table 2 for the whole population calculated by using linear and logistic regression models adjusted for age, sex, number of teeth, smoking, and status of marginal periodontitis. The estimates are presented for a 10-fold increase in the antibody or bacterial levels, number of root canal-treated teeth associated with saliva IgA and IgG against bacteria, and cross-reacting IgG. Among these, only saliva IgG against bacteria associated with the presence of root canal-treated teeth with an OR (95% CI) 2.52 (1.43–4.43). Number of widened periapical spaces associated with saliva cross-reactive IgA and IgG, saliva IgG against bacteria, and subgingival bacteria in linear regression models. The presence of widened periapical spaces associated with saliva IgA and IgG against bacteria with ORs 2.09 (1.01–4.34) and 2.25 (1.40–3.61), respectively, and with cross-reacting IgG, 1.56 (1.22–1.99). Significant associations were also observed between the presence of widened periapical space and Gram-positive and Gram-negative subgingival bacteria with ORs 1.40 (1.02–1.92) and 1.45 (1.05–2.00). Number of teeth with apical periodontitis associated with saliva IgG against bacteria and cross-reacting IgG, which also presented significant ORs with the presence of apical periodontitis (2.90 (1.71–4.92) and 1.62 (1.24–2.11)).

**Figure 3:**
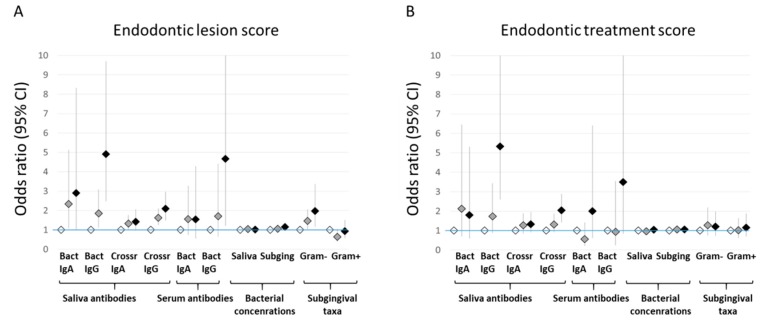
Associations of the antibody and bacterial levels with endodontic lesion score and endodontic treatment score. Endodontic lesion score (A): score I, no endodontic lesions; score II, patients with ≥1 widened periapical space and/or 1 tooth with apical periodontitis; and score III, patients with ≥2 teeth with apical periodontitis. Endodontic treatment score (B): score I, no endodontic lesions; score II, apical periodontitis only in teeth with root canal fillings; score III, apical periodontitis in teeth without root canal fillings. The associations were investigated by using multinomial regression models adjusted for age, sex, marginal periodontitis (healthy, gingivitis, periodontitis), number of teeth, and smoking (never/ever). The estimates for lowest (reference), middle, and highest scores are depicted with white, gray, and black diamonds, respectively.

A 3-category endodontic lesion score was designed for the severity of apical periodontitis. In addition, a 3-category endodontic treatment score was designed to investigate the contribution of treatment (root canal fillings) on the associations. The characteristics of the population are presented according to these scores in Table 3. Number of teeth, carious teeth, teeth with root canal fillings, and inadequate root canal fillings increased significantly with increasing scores. Also marginal periodontitis was more prevalent with high endodontic lesion (*p <* 0.001) or endodontic treatment (*p* = 0.287) score. Mean antibody levels in serum and saliva against specific antigens, as well as the saliva and subgingival bacterial levels according to these scores are presented in supplementary tables (Table S4 and S5). The association of the scores with the combined microbial biomarkers was analyzed by multinomial regression models for the log_10_-transforemed units (Figure 3). All measured parameters displayed positive trends with the increasing endodontic lesion score. Statistically significant associations (OR (95% CI)) with the highest endodontic scores were observed for saliva IgA (2.90 (1.01–8.33)) and IgG (4.91 (2.48–9.71)) against bacteria, saliva cross-reacting IgG (2.10 (1.48–2.97)), serum IgG against bacteria (4.66 (1.22–10.1)), subgingival species (1.15 (1.07–1.25)), and Gram-negative subgingival species (1.98 (1.16–3.37)). Regarding the treatment, only saliva IgG against bacteria, cross-reacting IgG, and serum IgA and IgG displayed increasing trends. Significant odds (OR (95%CI)) for untreated apical periodontitis were observed for saliva IgG against bacteria (5.32 (2.61–10.8)) and for cross-reacting IgG (2.04 (1.44–2.88)) (Figure 3).

The main results were reanalyzed in the subgroup of patients without marginal periodontitis (*n* = 132). There were no significant differences in the bacterial levels between groups divided according to the endodontic findings. Saliva IgG antibodies against bacteria and cross-reacting antibodies were higher in subjects with root canal fillings (*p* = 0.003 and 0.004), widened periapical spaces (*p* = 0.008 and 0.008), and apical periodontitis (*p* = 0.012 and 0.385). Both antibody levels increased in groups of patients with increasing endodontic scores (p for linear trend 0.009 and 0.020). The antibodies against bacteria (*p* for linear trend 0.007), but not the cross-reacting antibodies (*p* = 0.569), increased in patients with greater endodontic treatment scores. The associations of the saliva IgG class antibodies with endodontic lesion score and endodontic treatment score are presented in Table 4. Increasing trends were observed clearly only for saliva IgG against bacteria; the multivariate odds (OR (95% CI)) for having multiple teeth with apical periodontitis and for having teeth with untreated apical periodontitis were 3.45 (0.83–14.3) and 4.77 (1.05–21.7), respectively.

## 4. Discussion

We showed that apical periodontitis is associated with elevated levels of saliva IgA and IgG and serum IgG against bacterial antigens and saliva cross-reacting IgG, which recognise both bacterial and host epitopes. The associations were independent of marginal periodontitis. The local antibody response may contribute to the systemic IgG levels, which associate with the severity of apical periodontitis and arise mainly from untreated apical infections. High salivary IgA was associated with the number of widened periapical spaces, most likely indicating early endodontic infection.

Elevated levels of salivary total IgG associated with endodontic findings including root canal treatments, widened periapical spaces and radiographically diagnosed apical periodontitis. Both the presence and number of endodontic findings were significantly associated with total salivary IgG levels. In the case of total salivary IgA, the presence of root canal fillings and the number of widened periapical spaces, but neither the presence nor the number of teeth with apical periodontitis, associated with higher antibody levels. In health, the saliva IgGs mainly derive from the circulation by transudation through the gingival crevice. They comprise less than 15 percent of the total salivary immunoglobulins, as the major salivary immunoglobulin is secretory IgA produced by the salivary glands in mucosal plasma cells [38,39]. However, high concentrations of IgG and IgA and smaller amounts of IgM and secretory IgA have been detected within the periapical granulomas, in periapical cysts, as well as in root canal exudates with periapically affected teeth [40]. In addition, the total IgG and IgA levels detected from the periapical exudate were shown to correlate with clinical findings of the infected teeth [41]. As the half-life of IgA-class antibodies is only a few days, they are considered to reflect either recent or repeated exposure to the pathogen, while IgG is more stable, thus indicating a past, and maybe chronic, infection. Widened periapical spaces reflect either symptomatic teeth with irreversible pulpitis or precursors for established AP in necrotic teeth [1]. Since all determined antibody levels and bacterial concentrations correlated with the endodontic score, our results support the suggestion that the widened periapical spaces are likely to reflect early endodontic lesions [20].

When antibody response was studied in more detail, it was observed that salivary IgG levels against all studied species (*A. actinomycetemcomitans*, *P. gingivalis*, *P. intermedia*, *P. endodontalis*, and *T. forsythia)* were significantly higher in the groups with endodontic findings. In addition, patients with widened periapical spaces had higher saliva IgA-antibodies against *P. endodontalis*. It is widely accepted that endodontic infections have a multimicrobial etiology [4], and several pathogens associated with marginal periodontitis, such as *P. intermedia*, *P. gingivalis*, *T. denticola* and *P. endodontalis*, are frequently detected in teeth with necrotic pulps [42,43,44]. As apical periodontitis is often restricted to the periapical tissues, it is not surprising that the amount of studied salivary and subgingival bacteria were not consistently associated with the endodontic findings. On the other hand, it is reported that in the case of combined endodontic-periodontal lesions, where apical periodontitis can be initiated either in the pulp or in the periodontium, the microbial profiles of apical lesions and periodontal pockets resemble each other [10]. In such cases, it is probable that bacteria enter the root canal from the periodontium via the apical foramen, dentinal tubules and accessory root canals [45].

Two major pathogens in marginal periodontitis, *A. actinomycetemcomitans* and *P. gingivalis*, express several virulence factors including *P. gingivalis*-specific gingipains degrading the extracellular matrix and bioactive peptides [46], as well as heat shock proteins (HSPs) produced by both species [47]. These proteins elicit strong antibody production and are also able to induce a variety of cross-reactive antibodies recognizing human epitopes such as HSPs and oxidized low-density lipoproteins (oxLDL). These cross-reactions are considered potential links between periodontitis and an increased risk of cardiovascular diseases [21]. The oxidation of LDL gives rise to various epitopes and a frequently used model of oxLDL include the immunodominant epitopes malondialdehyde (MDA) and malondialdehyde acetaldehyde (MAA). It is reported that the presence of antibodies binding to MDA-LDL is associated with both the progression of atherosclerosis and with the presence and severity of periodontitis [37,48,49,50]. A monoclonal IgM antibody to MDA-LDL recognizes *P. gingivalis* virulence factor gingipain (Rgp44) as an antigen [34] and *A. actinomycetemcomitans* heat shock protein 60 (Aa-HSP60) cross-reacts with MAA-LDL [35]. To our knowledge, this study is the first to show the association between apical periodontitis and salivary cross-reactive antibodies. Especially cross-reactive antibodies representing IgG-class were strongly associated with different endodontic conditions.

All measured parameters displayed positive trends with increasing number of endodontic findings as both the salivary IgA and IgG against bacterial antigens, as well as the cross-reacting IgG, were significantly associated with the highest endodontic score. In addition, the effect of endodontic treatment on antibody response was evident indicating that the levels of both saliva IgG against bacterial antigens and cross-reacting IgG were significantly higher in the patients with primary apical periodontitis compared to those who had apical periodontitis in teeth with root canal fillings. The aim of root canal treatment is to eradicate the biofilm from the infected root canal and prevent the recurrent infection. However, if the treatment is inadequately performed, some bacteria may survive and cause secondary infection. The microbiome of endodontically treated root canals consists of fewer bacterial species, and some of the species are more resilient to endodontic treatment [10]. This phenomenon may reflect to the levels of antibodies measured in this study.

The production of local IgGs is also enhanced in advanced marginal periodontitis by local plasma cells of the gingiva [51]. We repeated our main analyses in a subgroup of subjects without marginal periodontitis. Although the number of patients in this subgroup was low, the association between saliva IgG against bacterial antigens and primary apical periodontitis remained significant, suggesting further that IgG antibody response is independent of marginal periodontitis.

The main limitation of this study is that our study population consists of middle-aged and elderly participants, and thus the oral infections are very common. In addition, all participants had an initial indication for coronary angiography. Another restriction is the lack of intracanal bacterial samples; hence the bacterial analyses were only conducted from saliva and subgingival samples. Different methods were used for the detection of the antibody levels in serum and saliva, and not the same antibody panels were available. For instance, we did not have information on the serum cross-reactive antibodies, which will be an aim for future investigations. Also different methodologies were used for the bacterial analyses, since the subgingival samples were examined by checkerboard DNA–DNA hybridization and the saliva samples by qPCR.

Although apical periodontitis has been considered as a potential risk factor for systemic diseases such as coronary artery disease (CAD) [52], only a few studies have attempted to draw conclusions on the associations between apical periodontitis and systemic diseases. Evidence for the association between AP and CVDs, such as endothelial dysfunction [53], atherosclerosis [54], and coronary heart disease [55], has been reported in separate studies. However, recent systematic reviews suggest only modest participation of endodontic infection on the systemic levels of biomarkers and a moderate or low correlation between some systemic diseases and apical periodontitis [14,23,24,56]. In our recent study, we demonstrated a confounder-adjusted association between apical periodontitis and CAD [20]. In the present study, we showed that apical periodontitis may contribute to the levels of IgG in serum which link oral bacteria to CAD risk [31]. These serum antibodies have been repeatedly associated with prevalent and incident CVD as well as with subclinical atherosclerosis [57,58,59,60]. Also the saliva cross-reacting antibodies and immunoglobulins against bacterial antigens have been associated with increased risk for CAD [37].

Salivary immunoglobulins are potential biomarkers of oral infectious diseases, but the specific antigens should be selected carefully. This would be especially beneficial in the case of apical periodontitis, as the disease is often asymptomatic and remains undiagnosed. This study represents a limited set of antibodies against selected bacterial targets, and further research is needed to investigate the levels of antibodies against other bacterial species commonly found in infected root canals.

## 5. Conclusions

Our results suggest that the inflammatory condition caused by endodontic infections could be identified by the increased salivary IgG levels independently of marginal periodontitis. The levels of saliva IgG may have a small, but significant effect on the systemic levels of biomarkers, indicating the potential link between apical periodontitis and systemic diseases.

## Figures and Tables

**Table 1:** Characteristics of the population.

Character		Mean (SD)
Age (years)		62.9 (9.1)
BMI (kg/m^2^)		27.8 (5.1)
Number of teeth		21.4 (7.5)
		**Mean (95% CI)**
Number of implants		0.12 (0.05–0.18)
Carious teeth		0.99 (0.84–1.14)
Root canal fillings		2.17 (1.96–2.39)
Inadequate root fillings		1.08 (0.95–1.20)
Widened periapical space		0.80 (0.71–0.89)
Apical periodontitis		0.36 (0.27–0.45)
With root canal fillings Without root canal fillings		0.16 (0.12–0.21)
		0.19 (0.11–0.27)
		**N (%)**
Gender (males)		284 (67.0)
Smoking (ever)		220 (51.9)
Hypertension		266 (62.9)
Diabetes (type I or II)		92 (21.9)
Dyslipidemia		340 (80.6)
Carious teeth		198 (47.4)
Root canal fillings		304 (71.7)
Widened periapical spaces		224 (53.8)
Apical periodontitis		101 (23.8)
Endodontic lesion score	No endodontic lesions	162 (38.2)
	≥1 tooth with widened periapical space or one tooth with apical periodontitis	194 (45.8)
	≥2 teeth with apical periodontitis	68 (16.0)
Endodontic treatment score	No endodontic lesions	323 (76.2)
	Apical periodontitis in teeth with root canal fillings	51 (12.0)
	Apical periodontitis in teeth without root canal fillings	50 (11.8)
Marginal periodontitis	Healthy	42 (9.9)
	Gingivitis	61 (14.4)
	Periodontitis	320 (75.5)

**Table 2:** Associations of endodontic findings with saliva and serum antibody levels and bacterial concentrations.

	Root Canal Fillings		Widened Periapical Space		Apical Periodontitis	
	Beta, *p*-Value	OR (95% CI), *p*	Beta, *p*-Value	OR (95% CI), *p*	Beta, *p*-Value	OR (95% CI), *p*
Saliva IgA against bacteria *	**0.096, 0.041**	2.162 (0.934–5.004), 0.072	0.062, 0.204	**2.090 (1.007–4.337), 0.048**	0.059, 0.235	1.995 (0.870–4.573), 0.103
Saliva IgG against bacteria *	**0.169, <0.001**	**2.519 (1.431–4.434), 0.001**	**0.138, 0.005**	**2.246 (1.397–3.611), 0.001**	**0.182, <0.001**	**2.904 (1.714–4.922), <0.001**
Serum IgA against bacteria *	0.031, 0.527	1.144 (0.516–2.539), 0.741	0.017, 0.738	1.513 (0.753–3.040), 0.245	0.037, 0.476	0.922 (0.421–2.018), 0.838
Serum IgG against bacteria *	0.010, 0.838	1.272 (0.443–3.656), 0.655	0.002, 0.963	2.048 (0.840–4.995), 0.115	0.083, 0.102	1.710 (0.615–4.754), 0.304
Saliva pathogen sum	0.012, 0.802	0.996 (0.932–1.064), 0.900	0.068, 0.163	1.046 (0.989–1.107), 0.117	0.079, 0.111	0.998 (0.936–1.064), 0.951
Subgingival pathogen sum	0.053, 0.273	0.991 (0.934–1.051), 0.760	0.086, 0.085	1.051 (0.999–1.105), 0.056	**0.098, 0.049**	1.047 (0.987–1.112), 0.127
45 gram-positive taxa	0.025, 0.603	0.754 (0.515–1.104), 0.147	**0.121, 0.014**	**1.395 (1.016–1.917), 0.040**	0.045, 0.375	1.096 (0.761–1.578), 0.622
33 gram-negative taxa	0.056, 0.237	0.945 (0.660–1.353), 0.757	**0.121, 0.014**	**1.446 (1.046–1.999), 0.026**	0.059, 0.238	1.249 (0.850–1.836), 0.257
Saliva cross-reactive IgA **	0.086, 0.067	1.248 (0.926–1.680), 0.146	**0.096, 0.049**	1.295 (0.995–1.685), 0.055	0.064, 0.196	1.327 (0.986–1.788), 0.062
Saliva cross-reactive IgG **	**0.128, 0.006**	1.240 (0.942–1.632), 0.125	**0.160, 0.001**	**1.555 (1.217–1.987), <0.001**	**0.159, 0.001**	**1.615 (1.237–2.108), <0.001**

The dependent variable in the linear regression was the number of findings and in the logistic regression the presence of findings. Adjusted for age, sex, marginal periodontitis (healthy, gingivitis, periodontitis), number of teeth, and smoking (never/ever). * Antibodies against *A. actinomycetemcomitans*, *P. gingivalis*, *P. intermedia*, *P. endodontalis*, and *T. forsythia*; ** Cross-reactive antibodies, antigens Pg-Rgp44, Aa-HSP60, and MAA-LDL.

**Table 3:** Characteristics of the population according to endodontic scores.

Character	Endodontic Lesion Score				Endodontic Treatment Score			
	Score I	Score II	Score III		Score I	Score II	Score III	
	**Mean (SD)**			**P ^1^**	**Mean (SD)**			**P ^1^**
Age (years)	63.3 (9.2)	63.1 (8.7)	63.7 (9.8	0.924	63.3 (9.0)	64.3 (9.0)	62.5 (9.5)	0.578
BMI (kg/m^2^)	27.8 (5.0)	27.8 (5.1)	27.8 (4.4)	0.990	27.7 (5.0)	27.9 (4.8)	28.2 (4.8)	0.852
Number of teeth	18.1 (10.5)	21.5 (7.3)	22.0 (6.7)	**<0.001**	19.7 (9.3)	23.2 (6.0)	20.5 (8.0)	**0.031**
	**Mean (95% CI)**				**Mean (95% CI)**			
Number of implants	0.17 (0.04–0.29)	0.10 (0.01–0.20)	0.12 (0.04–0.28)	0.696	0.15 (0.06–0.23)	0.14 (0.07–0.35)	0.02 (0.02–0.06)	0.546
Carious teeth	0.68 (0.52–0.84)	0.97 (0.75–1.18)	1.74 (1.20–2.27)	**<0.001**	0.83 (0.69–0.98)	1.24 (0.76–1.71)	1.82 (1.10–2.53)	**<0.001**
Root canal fillings	0.95 (0.73–1.17)	2.54 (2.26–2.82)	3.91 (3.21–4.61)	**<0.001**	1.82 (1.61–2.02)	4.65 (3.84–5.45)	1.91 (1.28–2.54)	**<0.001**
Inadequate root fillings	0.41 (0.29–0.52)	1.26 (1.10–1.42)	2.07 (1.64–2.51)	**<0.001**	0.88 (0.76–1.00)	2.33 (1.83–2.84)	1.05 (0.65–1.44)	**<0.001**
	**N (%)**			**P ^2^**	**N (%)**			**P ^2^**
N (%)	189 (41.7)	196 (43.3)	68 (15.0)		352 (77.7)	51 (11.3)	50 (11.0)	
Sex (males)	123 (65.1)	132 (67.3)	47 (69.1)	0.803	234 (66.5)	33 (64.7)	35 (70.0)	0.842
Smoking (ever)	92 (48.9)	112 (57.1)	35 (51.5)	0.265	190 (54.1)	23 (45.1)	26 (52.0)	0.478
Hypertension	122 (64.6)	123 (63.4)	41 (61.2)	0.885	226 (64.6)	30 (58.8)	30 (61.2)	0.683
Diabetes (type I/II)	39 (20.9)	47 (24.2)	14 (21.2)	0.711	78 (22.4)	11 (22.0)	11 (22.4)	0.998
Dyslipidemia	148 (78.3)	165 (85.5)	49 (73.1)	0.050	285 (81.7)	42 (84.0)	35 (70.0)	0.121
Marginal periodontitis	111 (58.7)	154 (78.6)	56 (82.4)	**<0.001**	244 (69.3)	37 (72.5)	40 (80.0)	0.287

^1^ ANOVA; ^2^ Chi-square test; Endodontic lesion score: score I, no endodontic lesions; score II, patients with ≥1 widened periapical space and/or 1 tooth with apical periodontitis; and score III, patients with ≥2 teeth with apical periodontitis. Endodontic treatment score: score I, no endodontic lesions; score II, apical periodontitis only in teeth with root canal fillings; score III, apical periodontitis in teeth without root canal fillings.

**Table 4:** Association of saliva IgG class antibody levels with endodontic scores in the subpopulation free from marginal periodontitis.

		OR (95% CI), *P*-Value			
		Saliva IgG Against Bacteria *		Saliva Cross-Reacting IgG **	
		Univariate	Multivariate ^1^	Univariate	Multivariate ^1^
Endodontic lesion score	No endodontic lesions	1.0	1.0	1.0	1.0
	≥1 tooth with widened periapical space or apical periodontitis	**2.92 (1.17–7.26), 0.022**	**2.67 (1.03–6.94), 0.044**	**2.06 (1.31–3.22), 0.002**	**1.97 (1.24–3.11), 0.004**
	≥2 teeth with apical periodontitis	**3.88 (0.99–15.5), 0.050**	3.45 (0.83–14.3), 0.088	1.32 (0.65–2.69), 0.435	1.30 (0.64–2.62), 0.472
Endodontic treatment score	No endodontic lesions	1.0	1.0	1.0	1.0
	Apical periodontitis in teeth with root canal fillings	2.52 (0.70–9.06), 0.157	2.44 (0.66–9.06), 0.184	1.11 (0.60–2.03), 0.746	1.12 (0.60–2.08), 0.731
	Apical periodontitis in teeth without root canal fillings	**5.88 (1.32–26.2), 0.020**	**4.77 (1.05–21.7), 0.043**	1.43 (0.73–2.80), 0.297	1.39 (0.70–2.78), 0.351

^1^ Adjusted for age, sex, and smoking (never/ever). Multinomial logistic regression. * Antibodies against *A. actinomycetemcomitans*, *P. gingivalis*, *P. intermedia*, *P. endodontalis*, and *T. forsythia*; ** Cross-reactive antibodies, antigens Pg-Rgp44, Aa-HSP60, and MAA-LDL.

## Supplementary Materials

The following are available online at https://www.mdpi.com/2077-0383/8/6/889/s1, Table S1: Bacterial species determined from subgingival plaque, Table S2: Saliva and serum antibody levels according to endodontic findings, Table S3: Saliva and subgingival bacterial levels according to endodontic findings, Table S4: Saliva and serum antibody levels according to endodontic scores, Table S5: Saliva and subgingival bacterial levels according to endodontic scores.
